# Supplementary material for: Chromothripsis during telomere crisis is independent of NHEJ, and consistent with a replicative origin
Source: Genome Res. 2019 May;29(5):737–49. doi: 10.1101/gr.240705.118 (PMC6499312; doi:10.1101/gr.240705.118)
Supplement: Supplemental Material [file supp_gr.240705.118_Supplemental_file_1.zip › contigs/annotated_contigs/DB85/contig.2.DB85_length_659_mean_cov_8.13960546282.docx]

**DB85_length_659_mean_cov_8.13960546282**

```
ATTCCAGCTTCCAAAATGTTTTGCTATTTTTCTGTCTTCCATTTTGTGTACATATGCTTTAAAAAATAAAAACCCTTAAGGTCTATGTA
 >chr3:196598584-196598842 - E=1e-132 p=0e+00
TTTGACTGTATTTTTTTTTTTGGAGATGGAGTCTCACTGTGTCACCCAGGCTGGAGTGCAGTGACCTGATCTCGACTCACTGCAACCTC

CACCTCCCAGGTTCAAGAGATTCTCCTGCCTCAGCCTCCTGAGTAGCTAGGAGTACGAGTGTGTGCCACCATGC|ACACA|CTCTTTGT
                                                                                >chr3:1
CAGTGATTTTTTTTTTAGAGGAGTGTCGCTCTGTCGCCCAGGCTGCAGTGCAGTGGCGCCATCTCGGCTCACTGCAAGCTCTGCCTCCC
96593322-196593633 - E=1e-164
GGGTTCACGCCATTCTCCTGCCTCAGCCTCCTGAGTAGCTGGGACTACAGGCGTCCACCACCACGCTTGGCTAATTTTTTTTGTATTTC

TTTAGTAGAGACCGGGTTTCACCAAGTTAGCCAGGATAATCTCGATCTCCTGACCTCGTGATCTGCCCGCCTCAGCCCCCCAAAGTGCT

GGGATTACAGGCGTGAGCCACCGCACCCAG|AGATCGGAAGAGCGTCGTGTAGGGAAAGAGTGTAGGATAGGGTGTAGATCTCGGTGGT

CGCCGTATCATTAAAAAACCTACGCGAGGGCCCCACACT
```
